# Web-Based Interventions for Chronic Back Pain: A Systematic Review

**DOI:** 10.2196/jmir.4932

**Published:** 2016-07-26

**Authors:** Shashank Garg, Divya Garg, Tanvir C Turin, M Faruq U Chowdhury

**Affiliations:** ^1^ University of Calgary Department of Family Medicine University of Calgary Calgary, AB Canada

**Keywords:** Internet, chronic back pain, Web-based interventions, systematic review, cognitive behavioral therapy, empowerment, disability

## Abstract

**Background:**

Chronic low back pain is one of the most common presenting complaints to a physician’s office. Treatment is often challenging and recovery depends on various factors, often resulting in significant investments of time and resources.

**Objective:**

The aim of this review is to determine which Web-based interventions aimed at chronic low back pain are of benefit to patients.

**Methods:**

Randomized controlled trials (RCTs) studying Web-based interventions directed at adults with chronic low back pain were included. Retrospective studies, narrative reviews, nonrandomized trials, and observational studies were excluded. Electronic databases and bibliographies were searched.

**Results:**

In total, nine unique RCTs were identified (total participants=1796). The number of patients randomized in each trial ranged from 51 to 580. Four trials studied online cognitive behavioral therapy (CBT) and five trials studied other Web-based interventions with interactive features. Empowerment/control was improved in six studies. Use of CBT was associated with reduced catastrophization among patients. Mixed results were reported with regards to reduction in pain levels and disability, although some studies showed promise in reducing disability in the short term. One study that measured health care utilization reported reduced utilization with the use of moderated email discussion.

**Conclusions:**

Limited data are available regarding effective Web-based interventions to improve outcomes for patients with chronic low back pain. Nine RCTs with small sample sizes were identified in this review. Online CBT appears to show some promise in terms of reducing catastrophization and improving patient attitudes. Further research in this area with larger-scale studies focusing on appropriate outcomes appears to be a priority.

## Introduction

Low back pain is one of the most common presenting complaints in physicians’ offices in North America [1]. Annual incidence of this condition in adults has been estimated to be between 10% and 15% worldwide [1]. The 3-month prevalence of low back and/or neck pain has been reported to be as high as 31% in the Unites States [2]. In addition to affecting the patient’s physical and psychological well-being, there are many other ways this condition impacts the population’s health and society in general. Back pain is a common cause of disability, absence from work and loss of productivity [3]. Back pain has significant economic repercussions including loss of productivity, morbidity, and costs to the health care system [4]. For example, Americans spend at least US $50 billion per year on low back pain [5]. Further, multiple studies have shown that absence from work affects patient’s well-being negatively and an increased length of absence makes it less likely that the individual will return to work [6,7].

Although the prognosis for low back pain remains good if the pain resolves in the acute phase (less than 3 months), individuals unable to do so may face a slow recovery at significant cost to self and the health care system [1]. Researchers have demonstrated that the treatment of back pain is complex [8]. This is because etiology may be multifaceted. Patient factors include age, presence of chronic disease, comorbidities, obesity, and sedentary lifestyle. Environmental factors can include work duties that require tasks such as heavy lifting, ergonomics, and others. Research on the effectiveness of rehabilitation interventions shows mixed results. A recent systematic review [9] reports insufficient data to draw conclusions about the effectiveness of specific interventions including back schools, massage, and patient education.

Due to the complex nature of chronic low back pain, effective treatment may include use of a multidisciplinary team (MDT). A MDT may be composed of a number of professionals, including a kinesiologist, physiotherapist, psychologist, occupational therapist, and pharmacist. A recent review reports that intensive multidisciplinary rehabilitation improves function in chronic back pain [8]. However, intensive daily rehabilitation for periods of up to 6 weeks [8] would require a significant commitment on behalf of the patient and at significant financial cost. Many patients have several barriers to access health professionals including lack of time, financial coverage, and lack of understanding of their role. Chronic pain is also known to have negative effects on the patient’s propensity for “self-management” of their chronic condition. There is a need for treatment approaches that are easily accessible, cost-effective, and reduce the effort required on the part of patients.

Recently, there has been some interest in using the Internet as a channel to offer interventions to treat chronic low back pain. This has several advantages. Some of the barriers that apply to face-to-face meetings with medical professionals may be ameliorated through the Internet. For example, patients can use online resources at their own convenience and may be able to reduce their health care-related costs. It is possible that Web-based interventions may lead to patient empowerment by supporting ownership over their health thereby encouraging patients to be more proactive about the treatment, maintenance, and follow-up of their condition.

The purpose of this review is to summarize randomized controlled trials (RCTs) that assess the effectiveness of Web-based interventions to support patients with chronic low back pain.

## Methods

### Data Sources and Searches

Electronic databases were searched for relevant citations between January 2000 and September 2014, including MEDLINE, PUBMED, and EMBASE. Search terms included: “Internet based,” “Internet-based,” “Internet Delivered,” “Internet-Delivered,” “Web based,” “Web-based,” “World Wide Web,” “Online,” “Telemedicine,” “Tele-medicine,” “Email,” “E-mail,” “Mobile,” “Phone,” “Smartphone,” “Tablet,” “intervention,” “treatment,” “therapy,” “communication,” “counseling,” “education,” “educational,” “instruction,” “management,” “self management,” “chronic,” “recurrent,” “duration greater than 3 months,” “low back pain,” “low-back pain,” “lower back pain,” “lumbar,” “lumbosacral region,” “mechanical,” “degenerative disc disease,” “sciatica,” “myofascial back pain,” “nonspecific back pain,” and “adult”. Publication types and study designs of interest including systematic reviews, meta-analyses, practice guidelines, RCTs, and controlled clinical trials. Bibliographies of eligible articles were also searched for relevant studies. Selected journals were also searched individually for any relevant publications.

### Study Selection

Articles were eligible for inclusion in this review if they were RCTs studying Web-based interventions directed at adults with chronic low back pain. Retrospective studies, narrative reviews, nonrandomized trials, and observational studies were excluded. However, references listed in these publications were reviewed to look for any studies that may match inclusion criteria for this review. Trials including children or trials including patients with acute pain were also excluded. RCTs studying interventions aimed at prophylaxis or other types of chronic pain were excluded. Studies published in languages other than English were excluded.

**Figure 1:**
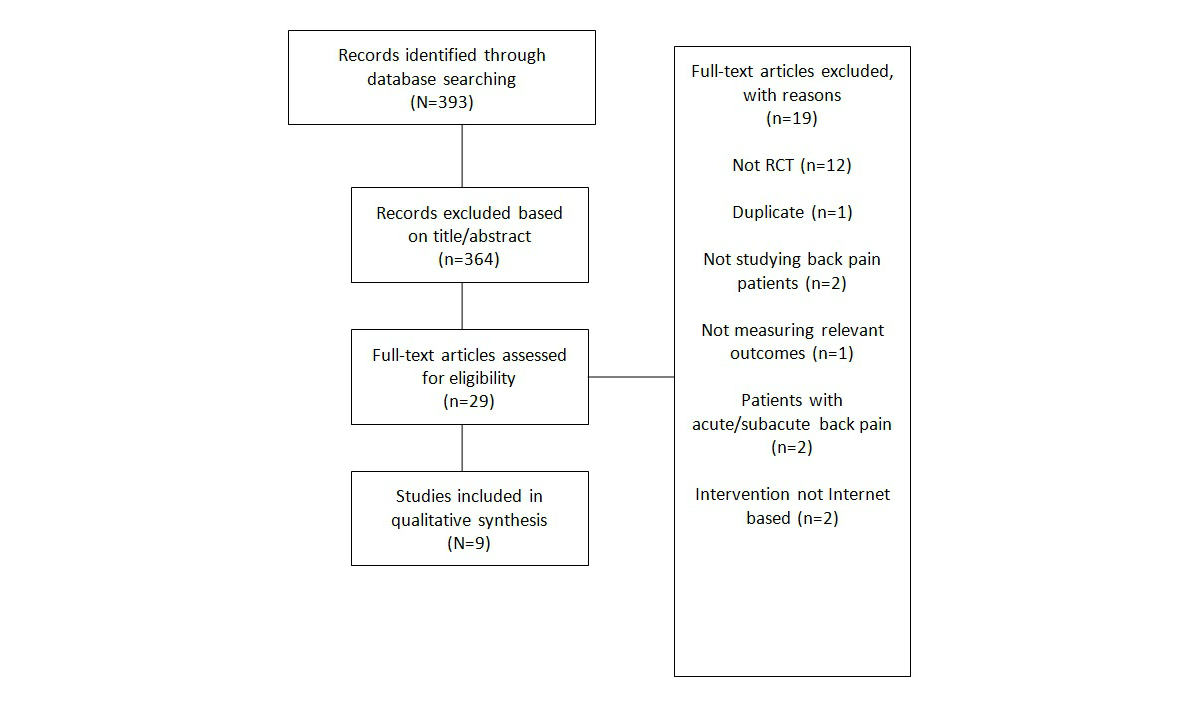
PRISMA flow diagram.

After the literature search identified potentially relevant articles, the articles were screened based on titles and abstract. Articles were excluded if they were not RCTs, the patient population was unsuitable for this review, the intervention was not Internet-based, or for other reasons (Figure 1). After this stage, the full text for the remaining articles was reviewed and nine were included for the purposes of this review.

### Data Extraction

Citations identified by the literature search strategy were screened for eligibility by two of the authors (SG, DG) and discrepancies were resolved using the opinion of the other authors. Information regarding the patient characteristics, intervention, duration, study characteristics, study design, and outcome measures was extracted from each eligible trial by one author (SG) and then reviewed independently by the other authors. Information required to assess the characteristics of studies was reviewed, including method of randomization, whether statistical analyses were performed by intention-to-treat, and allocation concealment [10-18].

## Results

### Literature Search Results

The search revealed nine RCTs published between 2002 and 2014. Table 1 describes the characteristics of each study; six of the studies performed intention-to-treat analysis, three of the studies did not describe randomization, and allocation concealment was documented in only four of the published studies.

Trial design and details regarding the interventions used in the studies are presented in Table 2. Studies randomized 51 to 580 participants [10-18]. Study durations lasted from 6 weeks to 1 year. Three of the studies were waitlist controlled.

Patient characteristics including demographics are listed in Table 3. The majority of participants in the studies using online CBT were females. The mean age of participants in the studies ranged between 42 and 52 years.

A variety of diverse outcome measures were used; outcome measures used by each study are available in Table 4. Studies using CBT reported catastrophization as an outcome measure. Most studies reported patient empowerment and pain levels as outcome measures. Disability was reported by only five studies. Only one study assessed impact of intervention on health care utilization.

**Table 1:** Study characteristics (N=9).

Study	Year	Randomization	Intention-to-treat analysis	Allocation	Lost to follow-up, n (%)
Lorig et al [10]	2002	Not described	Performed by using last known data	Unclear	159 (27.4)
Burhman et al [11]	2004	Not described	Not performed	Unconcealed	5 (9)
Chaiuzzi et al [12]	2010	Adaptive/stratified randomization	Yes	Unclear	10 (5)
Burhman et al [13]	2011	Webpage (random.org)	Yes	Performed through webpage	4 (7.4)
Carpenter et al [14]	2012	Random number table	Not performed	Unclear	23 (16.3)
Moessner et al [15]	2012	Not described	Yes	Unclear	4 (5.3)
Krein et al [16]	2013	Random number generator	Yes	Assignment of participants through automated email message	19 (8.2)
Riva et al [17]	2014	Random number generator (permuted block randomization method)	Yes	No face-to-face contact; no identifying information linked to patient assessment	0 (0)
Weymann et al [18]	2015	Simple computerized randomization procedure	Yes	Concealed random allocation automatically performed using software	180 (47)

**Table 2:** Trial design of included studies.

Study	Patients randomized	Intervention	Control	Duration	Measurement Time
Lorig et al [10]	580	Moderated email discussion group; back pain help book; videotape	Control group received usual care	1 year	Baseline, 6 and 12 months
Burhman et al [11]	56	Web-based multimodal pain management program (CBT, stretching and exercise); weekly submission of pain diaries; weekly telephone support	Waitlist	8 weeks	Baseline, 8-weeks and 3-months postintervention
Chiauzzi et al [12]	228	painACTION back pain website based on CBT and chronic pain management principles that provided tailored information to participants logging in twice weekly	Control group received copy of back pain help book	6 months	Baseline, 1, 3, and 6 months
Burhman et al [13]	54	Web-based multimodal pain management program based on CBT; no weekly telephone support	Waitlist	12 weeks	Baseline and 12 weeks
Carpenter et al [14]	141	Web-based wellness workbook	Waitlist	6 weeks	Baseline, 3 and 6 weeks
Moessner et al [15]	75	Intervention consisted of: individualized self-monitoring module, moderated Internet-based chat	Treatment as usual	15 weeks	Baseline, 115 and 202 days
Krein et al [16]	229	Intervention: pedometer with access to uploaded personal walking data, walking goals, feedback, participation in e-community	Enhanced usual care group also received pedometers but no access to walking goals or feedback	12 months	Baseline, 6 and 12 months
Riva et al [17]	51	RCT with two arms: intervention and control group	Intervention group received access to back pain management website with interactive features (virtual gym, action plan, testimonials, quiz game); control group also used website, but no interactivity	8 weeks	Baseline, 4 and 8 weeks
Weymann et al [18]	382 (chronic low back pain)	Web-based information system for patients which was tailored for individual needs and dialog based	Access to information through website without tailoring or use of dialogs	12 weeks	Baseline, first visit, and 3 months

**Table 3:** Patient characteristics of included studies.

Study	Patients randomized	Demographics	Inclusion criteria	Exclusion criteria
Lorig et al [10]	580	Control group: 61% male, mean age 45 years; intervention group: 62% male, mean age 46 years	One outpatient visit for back pain within last year	Continuous back pain for >90 days causing major activity intolerance; no physician visits for back pain in past year; receiving disability payments; red flag symptoms; planned back surgery; back pain due to systemic illness; pregnancy; unable to understand English
Burhman et al [11]	56	62.5% female; mean age 44.6 years (SD 10.4)	Age 18-65 years; access to Internet; previous contact with physician; lumbar/thoracic/cervical back pain; chronic pain ≥3 months	Wheelchair bound; planned surgery; cardiovascular disease
Chiauzzi et al [12]	228	67% female; mean age 46.14 years (SD 11.99)	Presence of back pain for ≥10 days, for ≥3 consecutive months; spinal origin of pain; English language fluency	Nonspinal medical or systemic conditions that explain the back pain; cervical pain without low back pain; psychiatric hospitalization within past year
Burhman et al [13]	54	68.5% female; mean age 43.2 years (SD 9.8)	Access to Internet; chronic pain ≥3 months duration	Planned surgery; wheelchair bound; cardiovascular disease
Carpenter et al [14]	141	83% female; mean age 42.5 years (SD 10.3)	Non-cancer-related back pain; duration ≥6 months; mean pain rating >4; access to Internet;	Age <40 years (applied after start of study); CBT within past 3 years; pain duration <6 months
Moessner et al [15]	75	Control group: 54.3% female, mean age 46.6 years (SD 7.7); intervention group: 57.5% female mean age 45.2 years (SD 10.2)	Age >18 years; prior multidisciplinary treatment for 1 week	Cancer-related pain; insufficient Language skills; treatment duration <1 week
Krein et al [16]	229	Control group: 86% male, mean age 51.9 years (SD 12.8); intervention group: 89% male, mean age 51.2 years (SD 12.5)	Persistent back pain; ≥3 months; self-reported sedentary lifestyle (<150 min of physical activity per week); Internet access	Inability to walk one block; pregnancy
Riva et al [17]	51	Control group: 50% female, mean age 51 years (SD 14.1); intervention group: 51.9% female, mean age 44 years (SD 13.6)	Age >18 years; back pain >3 months; Italian native speakers	Concurrent involvement in other study
Weymann et al [18]	382 (chronic low back pain)	Control group: 59.1% female, mean age 52.7 years (SD 13); intervention group: 58.5% female, mean age 52.2 years (SD 13.1)	Age >18 years; chronic back pain defined as pain almost every day for period >12 weeks; diabetes type 2	Age <18 years; duration of pain <12 weeks; lack of Internet access

**Table 4:** Outcomes of included studies.

Study	Outcome measures^a^	Catastrophization^b^	Empowerment/Control^b^	Pain^b^	Disability^b^
Lorig et al [10]	Pain (VNS); disability (RMQ); role function; health distress (MOS); health care utilization	NA	Increase	Increase	Increase
Burhman et al [11]	CSQ; MPI; PAIRS; HADS; pain diary; treatment credibility; satisfaction with treatment format	Increase	Increase	No effect	NA
Chaiuzzi et al [12]	BPI; ODQ; DASS; PGIC; CPCI-42; PCS; PSEQ; FABQ	Increase	Increase	No effect	No effect
Burhman et al [13]	CSQ; MPI; PAIRS; QOLI	Increase	No effect	No effect	NA
Carpenter et al [14]	Primary: SOPA; others: FABQ, NMRS, PCS, RMQ, SES	Increase	Increase	No effect	No effect
Moessner et al [15]	Pain intensity (NRS); SF-36; RMQ; KPD-38; Secondary: HADS (anxiety,; depression), general psychologic impairment	NA	NA	Increase	Increase
Krein et al [16]	Primary: RMQ, MOS; others: pain intensity, Fear-Avoidance Beliefs Questionnaire physical activity subscale	NA	Increase	No effect	Increase (6-month assessment); no effect (12-month assessment);
Riva et al [ 17]	Empowerment (PES); exercise; medication misuse; pain burden	NA	Increase	Increase	NA
Weymann et al [18]	heiQ; patient knowledge; decisional conflict; preparation for decision making	NA	No effect	NA	NA

^a^BPI: Brief Pain Inventory; CPCI-42: Chronic Pain Coping Inventory; CSQ: Coping Strategies Questionnaire; DASS: Depression Anxiety Stress Scale; FABQ: Fear-Avoidance Beliefs Questionnaire; HADS: Hamilton Anxiety and Depression Scale; heiQ: Health Education Impact Questionnaire; KPD-38: Clinical Psychological Diagnostic System; MOS: Medical Outcomes Study; MPI: Multidimensional Pain Inventory; NMRS: Negative Mood Regulation Scale; NRS: Numeric Rating Scale; PAIRS: Pain and Impairment Relationship Scale; PCS: Pain Catastrophizing Scale; PES: Psychological Empowerment Scale; PGIC: Patients’ Global Impression of Change Scale; PSEQ: Pain Self-efficacy Questionnaire; QOLI: Quality of Life Inventory; RMQ: Roland-Morris Disability Questionnaire; SES: Pain Self-efficacy Scale; SOPA: Survey of Pain Attitudes; VNS: Visual Numeric Scale.

^b^In intervention group. NA: not available.

The studies were presented in two subsections: studies using online cognitive behavioral therapy (CBT) and studies using Web-based approaches to improve knowledge (with an interactive component to provide coping support).

The following trials were registered: Burhman et al [13], Krein et al [16], and Riva et al [17].

### Studies Using Online Cognitive Behavioral Therapy

Psychological factors, such as depressed mood, negative beliefs, and somatization, have been shown to affect chronicity of pain and disability related to the pain [19,20]. This review identified four RCTs published between 2004 and 2012 that examined the effectiveness of Internet-based CBT as part of the treatment strategy for chronic back pain. The number of participants randomized in each study varied between 54 and 228. The majority of participants in all four studies were women (62.5%-83%).

Burhman et al [11] used Internet-based CBT in conjunction with telephone support to treat chronic back pain. The study reported that 95 participants would be required for a power of 80%; however, due to lower enrollment the study remained underpowered. The primary outcome measure was catastrophization, defined as the experience of irrationally thinking that something is far worse than it actually may be. This was measured as a subscale of the Coping Strategies Questionnaire (CSQ). The CSQ consisted of measurements of the following parameters: diverting attention, reinterpret pain sensations, coping self-statements, ignore pain sensations, praying or hoping, catastrophizing, increase activity level, control over pain, and ability to decrease pain. Patients were randomized to Web-based pain management or a waitlist control. The intervention group received access to weekly online CBT modules, guidance with physical activity and stretching exercises, and coping strategies over the course of 12 weeks. The intervention group also received weekly telephone calls that included discussion about participant goals, relaxation training advice, exercise guidance, and discussion on coping strategies. These calls occurred during the same period as the online intervention. The treatment group showed lower tendency to catastrophize and also reported better control over pain at 8 weeks. Due to significant follow-up through telephone calls, it is unclear how much of the treatment effect can be attributed to the online modality of treatment as opposed to the telephone-based support.

Burhman et al [13] performed a similar study to the one described previously, but without ongoing telephone support as part of the treatment plan. In all, 54 patients were randomized; it was reported that the study was underpowered to detect differences with conventional levels of confidence. The treatment group in this study also showed improved scores on the catastrophization subscale. Although this study did not include telephone support as part of the intervention, ongoing email support was provided to participants. Therefore, it is not entirely clear how much of the treatment effect can be attributed to the online CBT modules as opposed to the effect of email support. Along with reduction in catastrophization, the participants also reported improved control over pain with the intervention. Because the current paradigm of chronic pain management stresses the importance of maintenance or improvement in patient function, this may be seen as an important initial step toward achieving better self-efficacy and an improvement in the patient’s ability to understand and manage their own pain. However, further research would be useful to clarify whether this may indeed translate into improvements in pain and disability scores.

Carpenter et al [14] also studied an online self-help CBT intervention. The study included 141 participants who had back pain for more than 6 months and were older than 21 years of age. Over the course of three weeks, the treatment group used an online wellness workbook that included elements of CBT; the results reported an improved ability to self-manage pain. The treatment group reported decreased pain catastrophizing and a more positive outlook toward their disability. After week 3, the treatment group reported an improvement in their perceived ability to cope with their pain. Conversely, the participants in the control group were less confident about their ability to manage pain and were more likely to believe that they should avoid exercise. The study then allowed both groups to access the online workbook after the 3-week period and repeated their assessments for all the participants at 6 weeks. It was reported that the differences in the two groups were no longer apparent at 6 weeks, suggesting that access to the workbook successfully affected participants’ pain-related beliefs.

Chaiuzzi et al [12] compared an intervention group with access to a website (painACTION for back pain) designed on CBT self-management principles with a control group of participants provided with a back pain help book. Participants were recruited online and through a specialty pain clinic. The sample size was 228; sample size and power calculations were not reported. The intervention group received access to the CBT website and a weekly chat moderated by a therapist. Posttreatment follow-up at 3 and 6 months was performed. Overall, the intervention group reported reduced stress and improved coping, but pain and physical functioning were not affected significantly. However, in a subgroup of patients recruited online, pain levels did appear to be improved with the intervention compared to the control group.

In summary, four small RCTs reporting the effects of Web-based CBT for chronic back pain have been identified. All studies found reduced catastrophization in patients receiving online CBT.

Each of the trials used different measures to report pain levels. These measures included the Pain and Impairment Relationship Scale (PAIRS), Pain Self-efficacy Scale (SES), a pain diary, and self-reported pain levels for least, average, and worst pains. Of the studies that examined CBT, only Carpenter et al [14] used CBT as an adjunct to opioid therapy. None of the studies reported significant differences in pain severity.

### Studies Using Web-Based Approaches and an Interactive Component

Web-based interventions with interactive features are being increasingly studied for their potential role in the management of chronic diseases. The results from a recent review indicated Web-based interactive interventions for patients with a variety of chronic conditions may have a positive impact on patient empowerment and may facilitate enhanced physical activity [21].

The studies discussed in this section target knowledge about chronic low back pain by providing online resources and also provide support for coping through Web-based interactive features.

Lorig et al [10] performed an RCT to examine the impact of participation in email discussion groups; the outcomes of interest were health status and health care utilization. Study duration was 1 year and 580 participants were randomly assigned to treatment and control groups. The intervention group was enrolled in an email discussion group where various aspects of back pain were discussed with input from content experts. The content experts included a physician, physical therapist, and psychologist. This study used moderated email discussion; however, the topics for email discussion were mostly driven by participants and no specific predesigned content was provided to the participants. Further, the intervention group also received a back pain help book and a videotape modeling active living with back pain. No particular physical activity routine or exercise was suggested; rather, the email discussion answered general questions raised by the participants. The control group did not receive any specific back pain treatment or advice. The study included a 6-month and 1-year follow-up. At 1 year, improvements in pain, disability, role function, and health distress were reported with the intervention. The study was powered to detect these differences with a significance of *P*<.05. Health care utilization was reduced in the treatment group, but not to a statistically significant degree. The number of physician visits were decreased in the treatment group. Further, the mean number of hospital days (back-related days of hospitalization) were reduced by 0.25 days for the intervention group as compared to an increase of 0.04 days for the control group. Self-care orientation was improved with treatment. The study also reported that older age was associated with greater disability. The authors indicate and recognize that there are multiple factors affecting pain levels and health care utilization due to chronic back pain; consequently, it is unclear how the results can be attributed to the various parts of the intervention (discussion group, back pain help book, and videotape).

Moessner et al [15] studied aftercare intervention for patients who had already received multidisciplinary therapy for back pain. The study randomized 75 patients; low power was reported due to small sample size. Participants received an Internet-based aftercare intervention lasting 15 weeks or treatment as usual. The aftercare program included an individualized online self-monitoring module, where participants answered questions about their compliance with appropriate health behaviors. Also, the aftercare consisted of a 90-minute weekly text-based chat for a period of 15 weeks. The chat was moderated by an experienced group therapist; session topics were decided by the therapist. A physician or physiotherapist were not included as moderators for this chat. The results reported improvements in disability with the intervention. No significant difference in depression or anxiety was reported. Despite the positive results, there is an important caveat: a significant amount of data was lost because only 34 of 75 patients completed all three assessments. Moreover, the authors did not report the components of the multidisciplinary rehabilitation; as such, there is no way of knowing whether—and the degree to which—results of their Internet-based intervention were affected by components of rehabilitation. The authors theorized that patient beliefs about chronic pain may have impacted follow-up.

Krein et al [16] conducted a study that focused on improving the activity level of the participants by providing them with pedometers that gave online feedback regarding their daily activity. The pedometer feedback was used in combination with an e-community social support group. The researchers randomized 229 patients into a control group and a treatment group. The study was designed to detect a clinically meaningful difference (0.4 standard deviation or 2-point difference) in Roland-Morris Disability Questionnaire score and sought to enroll 130 participants in each group to account for potential of 25% attrition; high rates of participant follow-up were achieved for this study allowing for detection of differences in primary outcome. The treatment group received pedometers along with access to online feedback including the number of steps and individual goals to promote improvement. The treatment group was also provided with access to an online social support group. In contrast, the control group received pedometers but did not receive online feedback or social support. Assessments were performed at 6- and 12-month time points. Most participants were males. Significant improvement was reported for the treatment group compared to the control group for back pain disability at 6 months, but the difference was no longer statistically significant at 12 months. No difference was reported between the groups in terms of Fear-Avoidance Beliefs Questionnaire Physical Activity subscale. Physical activity measured by step counts was increased in the intervention group at 6 months; however, this difference was less marked when measured at 12 months. Exercise self-efficacy scores were similar between the two groups at 12 months.

Riva et al [17] randomized 51 patients into two groups. It was reported that the study was designed to achieve power of 80% with 95% confidence, and sufficient numbers were recruited for this purpose. The intervention group received access to a self-management website with interactive components including quizzes, virtual gym, an action plan, and additional online resources. The control group only received access to static features and information on the website. Four- and 8-week assessments were performed. Outcome measures included empowerment, medication misuse, physical exercise, and pain burden. The intervention group was reported to have improved patient empowerment and reduced medication misuse. Pain burden decreased, but to equal measures in both the control and intervention groups. Because pain levels decreased in both groups, it appears that the interactive features available to the intervention group did not make a significant difference to their pain levels. However, participant empowerment was reported to be significantly improved in the intervention group. It appeared that interactivity and feedback through the Internet may improve a sense of control or empowerment in chronic back pain patients.

Weymann et al [18] included participants with chronic low back pain and type 2 diabetes in their study. A total of 561 participants were randomized, of which 382 were enrolled with chronic back pain. The intervention was a tailored interactive health communication app, which provided support to participants with regards to their knowledge and attitudes about their condition. The coping style of participants was assessed prior to intervention; participants in the intervention group were offered tailored content based on their coping style. For chronic low back pain participants, information was based on recent guidelines and Cochrane reviews. Primary outcomes were patient knowledge and patient empowerment.

The study aimed to detect differences with conventional levels of confidence and 80% power; however, due to attrition, only 202 of 382 chronic low back pain participants performed the 3-month follow-up. No significant differences were detected in outcomes with the intention-to-treat analysis.

Two of the studies [10,15] reported reduction in disability. Krein et al [16] reported reduction in disability at 6 months, which was not sustained in further assessments. Lorig et al [10] reported statistically significant reduction in pain, whereas Moessner et al [15] reported improvement with the pain subscale of the 36-item Short-Form Health Survey (SF-36), but not with Numeric Rating Scale (NRS).

Four of the studies did measure empowerment/self-efficacy and mixed results were reported. Empowerment was reported to be improved in one of the studies [17] and another study reported improved self-efficacy [10]. However, no difference was reported in other studies [16,18].

A variety of diverse outcome measures have been used in the studies. Lorig et al [10] did measure health care utilization, which was reported to be decreased in their treatment group. In the context of chronic low back pain, this outcome measure has not been extensively studied in RCTs since this trial; it would be prudent for future researchers to include cost or health care utilization as an outcome measure. Further, based on the study design, it may not be possible to ascertain the individual contribution of each part of the intervention to the reported outcomes. Also, the discussion is not supportive of any particular physical activity intervention and no individual medical advice was provided to participants. This suggests that participant self-efficacy may independently affect outcomes in this population.

## Discussion

Nine unique RCTs were identified addressing the impact of Web-based interventions on chronic low back pain. The major categories of interventions included online CBT and to improve knowledge with an interactive component to provide coping support. The trials identified had small sample sizes and many of them were not blinded. In terms of power calculations, three of the trials reported being underpowered. There is considerable concern with external validity for these study results. The demographics of the population included for the different studies were heterogenous. The delivery, format, and timeline of the interventions were also heterogenous. Most studies only reported posttreatment data and there is a lack of long-term follow-up. In the studies that do report longer-term data, the treatment effects seem to taper off with time [16].

Many of the studies excluded patients receiving disability payments, a significant part of the population that experiences chronic back pain. As such, the absence of research on this subpopulation is a major gap that should be addressed in future studies. The effect of Web-based interventions on health care utilization was reported by only one study [10] and indicated a trend toward reduced physician visits for back pain. This is an important outcome measure that would be useful to include in future studies to better understand effects of online interventions on health care access, system burden, and resources. CBT has been linked to improved outcomes in many chronic conditions and this review indicates that CBT has been effective for chronic pain. However, specific mechanisms through which the CBT treatment has its effect are not entirely clear and more research on this process is necessary [22]. Several studies have reported decreases in catastrophization and/or improvement in self-efficacy and this may lead to improvements in health-related behaviors or follow-up and adherence with appropriate treatments. Also, patient characteristics that make them more likely to respond to CBT have not been adequately studied [22].

Four RCTs reporting the effects of Web-based CBT for chronic back pain were identified for this review. Three of the studies report reduced catastrophization in patients receiving online CBT. In previous studies on chronic pain, catastrophization has been linked to increased severity of pain, poor treatment outcomes, and increased disability [1]. However, one limitation to the online CBT studies is that the majority of participants were women. Researchers have previously noted the women seek health care more often for pain compared to men [23]. Further, the incidence of low back pain appears to be higher among females and those aged between 40 and 80 years [24]. However, it is important to have studies with more male participants—or a mix of demographics—to improve the applicability and generalizability of the results. Also, the format and the dose of CBT provided in different trials are variable. Therefore, it is difficult to draw conclusions regarding the optimal frequency, duration, and format of CBT that may be required to improve outcomes in chronic back pain.

Further, there are various limitations to the studies using online CBT. All studies randomized small numbers of patients at single centers. Some of the studies are not adequately powered. One study was waitlist controlled, which can be problematic because this can make the results of the treatment effect appear more significant than it actually is. Intention-to-treat analysis was not conducted in two of the studies; therefore, participants with suboptimal compliance are excluded from parts of the analysis. Also, the form and type of delivery of supports in addition to online intervention were variable. For example, Burhman et al [11] made significant use of telephone support and provided consistent advice regarding physical activity, whereas Carpenter et al [14] focused solely on behavior and cognitive exercises. Therefore, effects found in Burhman et al [11] may be attributed to multiple interventions rather than CBT alone.

Another limitation is that most of the participants in the studies were females, which may affect the generalizability of the results. Some of the studies excluded patients with comorbidities such as heart disease; this may affect how results can be interpreted because many patients seen in practice with low back pain have significant comorbidities, which may also limit generalizability. In general, the samples in the studies may have been so carefully selected that their external validity is questionable.

Further, none of the studies included groups receiving face-to-face CBT as controls; therefore, it is not possible to estimate the efficacy of online CBT in comparison with the traditional approach. Overall, the research indicates that online CBT may be effective in reducing catastrophization and improving patient attitudes toward back pain, particularly when supported with telephone or email follow-up. However, additional RCTs with larger and more diverse samples are required to further investigate whether this intervention can be effective in reducing pain, disability, and health care costs. Furthermore, studies must be conducted to consider independent effects from total effects for each aspect of treatment.

Five RCTs reporting effects of Web-based approaches to improve knowledge and coping support. Three of these studies reported a reduction in disability [10,15,16], although in one study this benefit was not sustained on assessment at 1 year [16]. Two of the studies also appear to show improvement in pain levels [10,15]. Empowerment was reported to be improved in one of the studies [17] and another study reported improved self-efficacy [10].

These studies have a number of limitations. One omission is that Riva et al [17] did not include information on whether their samples were using medications to reduce their back pain over the course of their studies; however, it may be assumed that many patients with chronic back pain will access or use medications. In terms of patient empowerment and patient self-efficacy, mixed results were reported [16-18]. These two constructs are conceptually related; as such, we would expect to find similar effects of the online interactive intervention across both samples. Further, the studies have small sample sizes and three of the studies are underpowered. A majority of the participants in the study by Krein et al [16] were male, which can affect the generalizability of the results. Many of the studies have short duration and/or follow-up. Therefore, it may be difficult to draw conclusions regarding the long-term impact of the interventions, especially with regards to function and disability.

### Conclusions

Although research on many of the Web-based interventions for back pain reviewed here had mixed results or do not appear to have high external validity, we did find evidence that that there are likely some benefits to online CBT for reduced catastrophization. As such, online interventions may be a useful solution to overcome current limitations of traditional face-to-face CBT because, for example, access to professionals that are able to deliver high-quality CBT remains limited. Second, many patients may not be able to afford access to such professionals or counseling. Third, physical access may also be limited due to the nature of pain, patient comorbidities, or other social factors, and large geographical distances may preclude eligible patients from accessing specialized rehabilitation or chronic pain centers. Fourth, in some cases, there may be a stigma associated with the use of a therapist or counselor. Therefore, online access to CBT may help to alleviate some of the barriers to access and provide patients a convenient alternative to face-to-face visits. Future studies using CBT as an intervention should consider including appropriate numbers of male participants to improve the generalizability of the results.

Further, empowerment/control did show improvement in six of the studies. Three of these used CBT, whereas three of the studies used other forms of Web-based support, such as email/chat or other interactive features. It appears that forms of social support other than formalized counseling or CBT may have some positive effect on the patient’s ability to manage and cope with their chronic condition.

Disability was only assessed in five of the studies and mixed results were reported. Further research in this area with studies having longer follow-up should be a priority. One study reporting health care utilization reported positive effects with the intervention. It would be important for future studies to assess this further because it is important to focus resources on interventions that can reduce use of health care resources. Further research that includes these outcomes could provide insight into future planning for the health care system and implications for clinical practice.

## Abbreviations

BPI: Brief Pain Inventory
CBT: cognitive behavioral therapy
CPCI-42: Chronic Pain Coping Inventory
CSQ: Coping Strategies Questionnaire
DASS: Depression Anxiety Stress Scale
FABQ: Fear-Avoidance Beliefs Questionnaire
HADS: Hamilton Anxiety and Depression Scale
heiQ: Health Education Impact Questionnaire
MOS: Medical Outcomes Study
MDT: multidisciplinary team
MPI: Multidimensional Pain Inventory
NMRS: Negative Mood Regulation Scale
NRS: Numeric Rating Scale
PAIRS: Pain and Impairment Relationship Scale
PCS: Pain Catastrophizing Scale
PES: Psychological Empowerment Scale
PGIC: Patients’ Global Impression of Change Scale
PSEQ: Pain Self-efficacy Questionnaire
QOLI: Quality of Life Inventory
RCT: randomized controlled trial
RMQ: Roland-Morris Disability Questionnaire
SES: Self-Efficacy Scale
SOPA: Survey of Pain Attitudes
VNS: Visual Numeric Scale
